# Focal adenomyosis (intramural endometriotic cyst) in a very young patient - differential diagnosis with uterine fibromatosis

**Published:** 2016

**Authors:** L Manta, N Suciu, A Constantin, O Toader, F Popa

**Affiliations:** * Department of Gynecology and Obstetrics, “Gh. Polizu” Maternity, “Alfred Rusescu” Mother and Child Care Institute, Bucharest, Romania; ** Department of General Surgery, “Sf. Pantelimon” Clinical Hospital, Bucharest, Romania

**Keywords:** focal adenomyosis, uterine fibromatosis, uterine tumor, pelvic endometriosis

## Abstract

**Introduction.** Adenomyosis is a widespread disease usually affecting the late reproductive years of the women’s life, which has a great impact on their fertility. The most common form is diffuse adenomyosis, while focal adenomyosis, a cystic variant, is very rare, particularly in patients younger than 30 years old.

**Materials and methods.** We reported a rare case of a 20-year-old Caucasian woman with cystic adenomyosis who was admitted in our service with severe chronic pelvic pain, dysmenorrhea, and menorrhagia, who had received conservative surgical treatment to preserve fertility and improve her obstetrical prognosis.

**Results and Discussions.** Although the necrobiosis of a uterine fibroid was suspected preoperatively, the extemporaneous histopathological exam revealed adenomyosis associated with fibroleiomyoma with hyaline dystrophy and multiple foci of endometriosis of cystic formation in the wall of a young woman without any risk factors.

**Conclusion.** Although a rare lesion in young patients, cystic adenomyosis should be considered when chronic pelvic pain is exacerbated during menstruation and is associated with a uterine tumor. In young patients, the differential diagnosis should be made with uterine malformations (hematometra), necrobiosis of uterine fibroids, pelvic endometriosis. The surgical treatment should be conservative with the excision of the lesion, always taking into account fertility preservation in young patients.

## Introduction

Adenomyosis is a widespread disease usually affecting the late reproductive years of the women’s life, which has a great impact on their fertility. The most common form is diffuse adenomyosis, while focal adenomyosis, a cystic variant, is very rare, particularly in patients younger than 30 years old. It is characterized by the presence of ectopic endometrial tissue within the uterine myometrium, with adjacent smooth muscle hypertrophy [**1**]. The diagnostic criteria for juvenile cystic adenomyosis proposed by Hiroyuki et al. include severe dysmenorrhea, age < 30years and a cystic lesion ≥ 1cm in diameter independent of uterine lumen and covered by hypertrophic myometrium on diagnostic images [**2**].

## Case Report

We reported a case of a 20-year-old Caucasian woman who was admitted in our service complaining of chronic pelvic pain, exacerbated during the menstrual period and menorrhagia. The patient was nulliparous with no significant personal or family history, menarche at age 12, with a BMI of 19. She had a regular menstrual cycle with severe dysmenorrhea for years, which worsened in the past 3 months and treated with NSAIDs. Laboratory values disclosed no abnormalities on admission.

Physical examination revealed an increased uterine volume, similar to a 10 weeks old pregnancy, extremely painful during palpation, easily mobilized, with no other abdominal masses.

**Fig. 1:**
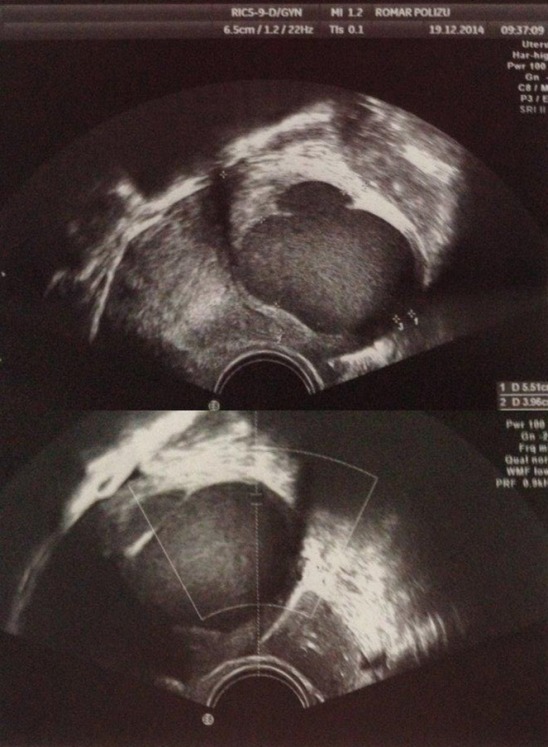
Preoperative color Doppler transvaginal ultrasound

Transvaginal ultrasound: uterus in AVF with long diameter of 6,48cm, anteroposterior diameter of 2.6cm, linearly endometrium with a thickness of 4mm. Within the anterior wall, intramurally, towards the uterine fundus, a well defined, round oval sized tumor formation of 3.97/ 4cm with a thin echogenic content, with no distortion or protrusion in the uterine cavity, without color Doppler signal within, was found (**Fig. 1**). No ovarian abnormalities were noted.

Preoperatively the necrobiosis of a uterine fibroid was suspected and a classic myomectomy with extemporaneous histopathological exam was decided to be practiced.

## Results

**Fig. 2:**
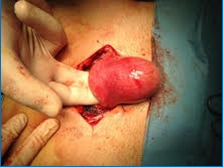
Intraoperative image showing the uterine tumor

**Fig. 3:**
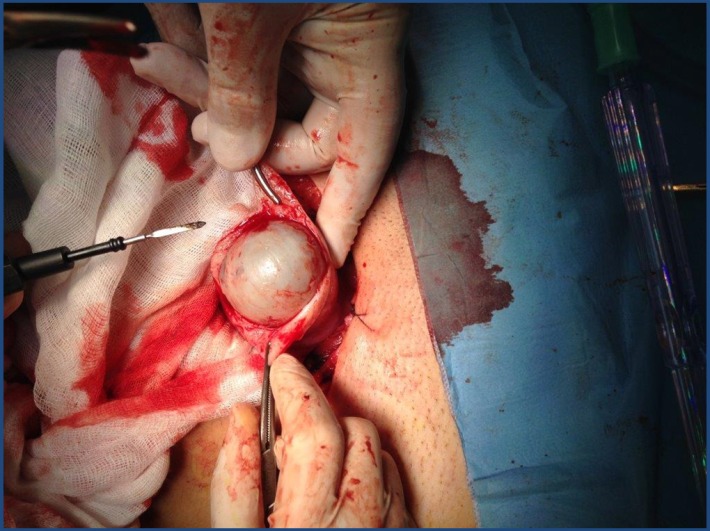
Intraoperative image showing the adenomyotic cyst aspect

**Fig. 4:**
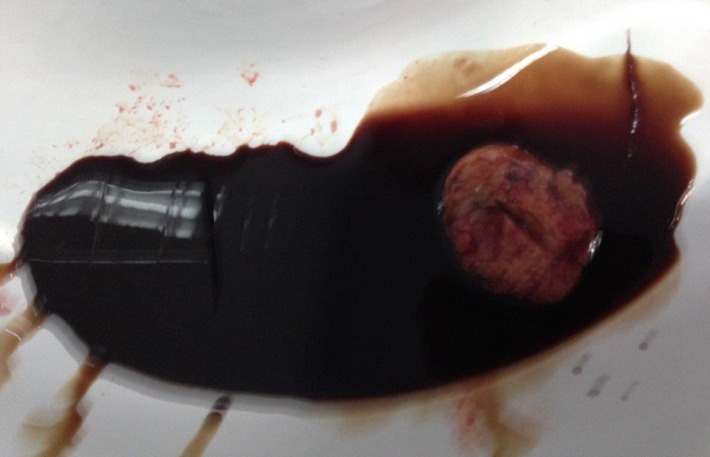
Sectioned uterine tumor and chocolate-like content

Intraoperatively, an increased uterine volume was found due to a 5cm nodule within the anterior wall without any visceral adhesions (**Fig. 2**). During excision, the nodule presented as a remitting cyst with white pearly walls and well defined cleavage planes (**Fig. 3**). When opening the cyst, a chocolate-like content highly suggestive of endometriosis was found, this being confirmed at the extemporaneous histopathological exam (**Fig. 4**). The intervention continued with a dual-layer myometrium suture with a favorable postoperative evolution. The histopathological examination revealed adenomyosis associated with fibroleiomyoma with hyaline dystrophy and multiple foci of cystic formation endometriosis in the wall.

## Discussions

Gynecological adenomyosis is a clinical challenge, due to the sometimes-difficult diagnosis that mainly occurs in multiparous women in perimenopause. Pregnancy seems to favor the development of adenomyosis in the myometrium due to the invasive nature of the trophoblast extension through the myometrial fibers [**3**,**4**]. In addition, the adenomyotic tissue seems to have an increased sensitivity to estrogen, so increased levels of estrogen in pregnancy would promote the development of this condition. Uterine curettage abortions and births by caesarean section also represent risk factors [**3**,**5**].

Although it has not been demonstrated, the causal link between a history of uterine surgery and adenomyosis should be taken into account. Smoking appears to be a protective factor for adenomyosis, which is explained by the lower estrogen serum levels between heavy smokers, knowing that adenomyosis, endometriosis, and uterine fibroids are estrogen-dependent diseases [**3**-**5**]. Adenomyosis development favors the appearance of intramural ectopic pregnancies and increases the risk of uterine rupture during pregnancy [**6**].

Depression and antidepressant therapy that interferes with the prolactin metabolism, promotes the progression of endometriosis and adenomyosis, acting as a mitogen at the myometrial and endometrial level. As estrogen dependent disease, uterine fibroids can coexist in 15-57% of the cases of adenomyosis, as demonstrated by the histopathological examination in our case [**3**,**7**,**8**]. Although uterine fibroids and adenomyosis have mutual symptoms like pelvic pain, dysmenorrhea, uterine bleeding and dyspareunia, studies have shown that they are more pronounced in women with adenomyosis than in those with fibromatosis. Uterine volume in adenomyosis is generally smaller than in uterine fibroids [**9**,**10**].

In this case, the patient was nonsmoker, infertile, nuligesta, nulliparous, with a normal BMI, so, none of the risk factors for the development of adenomyosis was present. Although adenomyosis and endometriosis have different etiopathogenesis, they have a similar pathological substrate, namely, the ectopic endometrial tissue [**3**,**4**]. In this case, even if the ectopic endometrial tissue location was that of adenomyosis, the risk factors and the evolution have approached the case pathophysiology and clinics more to endometriosis. The main differential diagnosis was established after a clinical examination with uterine fibroids, a more common pathology in young patients, because of the similar symptoms and the increased uterine volume. Although the ultrasound appearance was suggestive of intramural endometriosis (adenomyosis), the rarity of this disease in young patients, made the diagnosis difficult preoperatively, a necrobiosis of a uterine fibroid being suspected. Postoperatively, the patient reported pain symptoms, dyspareunia and menometrorrhagia amendment. The patient did not show any sign of pregnancy so far.

## Conclusions

Although a rare lesion in young patients, cystic adenomyosis should be considered when chronic pelvic pain is exacerbated during menstruation and is associated with a uterine tumor. The differential diagnosis in young patients should be made with uterine malformations (hematometra), necrobiosis of uterine fibroids, pelvic endometriosis. The surgical treatment should be conservative with the excision of the lesion, always taking into account fertility preservation in young patients. The reproductive outcome after a conservative surgical excision of cystic adenomyosis seems to be good.

**Acknowledgement**

This paper is supported by the Sectoral Operational Programme Human Resources Development (SOP HRD), financed from the European Social Fund and by the Romanian Government under the contract number POSDRU/187/1.5/S/156069/
